# Prediction and Modeling of Protein–Protein Interactions Using “Spotted” Peptides with a Template-Based Approach

**DOI:** 10.3390/biom12020201

**Published:** 2022-01-25

**Authors:** Chiara Gasbarri, Serena Rosignoli, Giacomo Janson, Dalila Boi, Alessandro Paiardini

**Affiliations:** 1 Dipartimento di Scienze Biochimiche “A. Rossi Fanelli”, Sapienza Università di Roma, 00185 Rome, Italy; c.gasbarri@celeristx.com (C.G.); serena.rosignoli@uniroma1.it (S.R.); dalila.boi@uniroma1.it (D.B.); 2 Department of Biochemistry and Molecular Biology, Michigan State University, East Lansing, MI 48824, USA; jansongi@msu.edu

**Keywords:** PepThreader, protein–protein interactions, protein–peptide interactions, template-based modeling

## Abstract

Protein–peptide interactions (PpIs) are a subset of the overall protein–protein interaction (PPI) network in the living cell and are pivotal for the majority of cell processes and functions. High-throughput methods to detect PpIs and PPIs usually require time and costs that are not always affordable. Therefore, reliable in silico predictions represent a valid and effective alternative. In this work, a new algorithm is described, implemented in a freely available tool, i.e., “PepThreader”, to carry out PPIs and PpIs prediction and analysis. PepThreader threads multiple fragments derived from a full-length protein sequence (or from a peptide library) onto a second template peptide, in complex with a protein target, “spotting” the potential binding peptides and ranking them according to a sequence-based and structure-based threading score. The threading algorithm first makes use of a scoring function that is based on peptides sequence similarity. Then, a rerank of the initial hits is performed, according to structure-based scoring functions. PepThreader has been benchmarked on a dataset of 292 protein–peptide complexes that were collected from existing databases of experimentally determined protein–peptide interactions. An accuracy of 80%, when considering the top predicted 25 hits, was achieved, which performs in a comparable way with the other state-of-art tools in PPIs and PpIs modeling. Nonetheless, PepThreader is unique in that it is able at the same time to spot a binding peptide within a full-length sequence involved in PPI and model its structure within the receptor. Therefore, PepThreader adds to the already-available tools supporting the experimental PPIs and PpIs identification and characterization.

## 1. Introduction

Protein functions are primarily based on their ability to establish interactions both with other proteins and other biomolecules [1,2,3,4]. Since protein–protein interactions (PPIs) play a key role in cell complexity and functionality, there is considerable interest in this field and, as a consequence, an ever-increasing availability of different methods and approaches for such studies. In parallel, experimental methods used to investigate protein–peptide interactions (PpIs) are similar to those used in PPIs studies and can be divided into three main classes: atomic-resolution approaches (crystallography [5,6], NMR [7], cryo-EM [8]), mass spectrometry [9,10], and other biochemical/biophysical methods [11,12,13,14]. Large-scale analysis, which is possible thanks to the synthesis of stabilized peptides [15,16,17] and the use of peptide arrays [18], has boosted peptide-based studies. Such information, in turn, can be used for drug design purposes, e.g., to design peptide derivatives able to hamper the interactions of therapeutic targets, also known as peptidomimetics [19]. However, the rate limiting step of in vitro PPIs and PpIs characterization is often the expression and purification of proteins involved in the interaction: many are insoluble or toxic when expressed in hosts, or they cannot be obtained in a great amount. For this reason, computational predictions represent a valid and effective strategy supporting the characterization of PPIs/PpIs by experimental means.

The high-resolution prediction of PpIs always requires the modeling of complex structures. Ab initio computational docking is often used for this purpose. Among the different docking methods developed so far [20,21,22], Rosetta FlexPepDock [23] and HADDOCK [24,25] predict complexes from an extended peptide and a given binding site, using local docking algorithms and optimization. An alternative, template-based approach, e.g., GalaxyPepDock [26], exploits the information derived from the increasing number of PpIs deposited in the Protein Data Bank (PDB; [27]), to generate high-resolution PpI complex models. When applicable (that is, when a structural template is highly similar to the investigated complex), the accuracy of template-based approaches, as assessed by the CAPRI (Critical Assessment of Predicted Interactions) project [28], often outperforms ab initio PpI prediction methods [29,30,31].

However, it must be noted that in all the mentioned cases, the identity (i.e., its sequence) of the peptide involved in the interaction must be known. Indeed, traditionally the main challenges lying ahead of a successful PpIs prediction are represented by the so-called “flexibility” and “scoring” problems [32]. The former is related to the huge number of possible conformations that the protein and peptide can have upon binding and induced fit. The latter is due to the difficulty of correctly ranking the possible solutions. On the other hand, a third related issue, perhaps overlooked to date and hereinafter named the “spotting” problem, is the ability to identify (“spot”) the peptide fragment of a full-length protein binding to a second receptor, and at the same time simulate its conformation and interaction. This challenging issue can be considered at the crossroads between PPIs and PpIs prediction and modeling. In an attempt to fill this structural gap, a computational tool is presented that is able to cope with the “spotting” problem, using an efficient template-based approach that requires short times of analysis. This program, called PepThreader, receives as input a full-length target sequence and gives as output a pool of peptide fragments that could possibly bind a receptor partner, providing also 3D models of the predicted protein–peptide complexes. To this end, the template-based approach of PepThreader makes use of a 3D structure containing the same receptor protein, in complex with a peptide similar to the target.

## 2. Methodology

**Figure 1:**
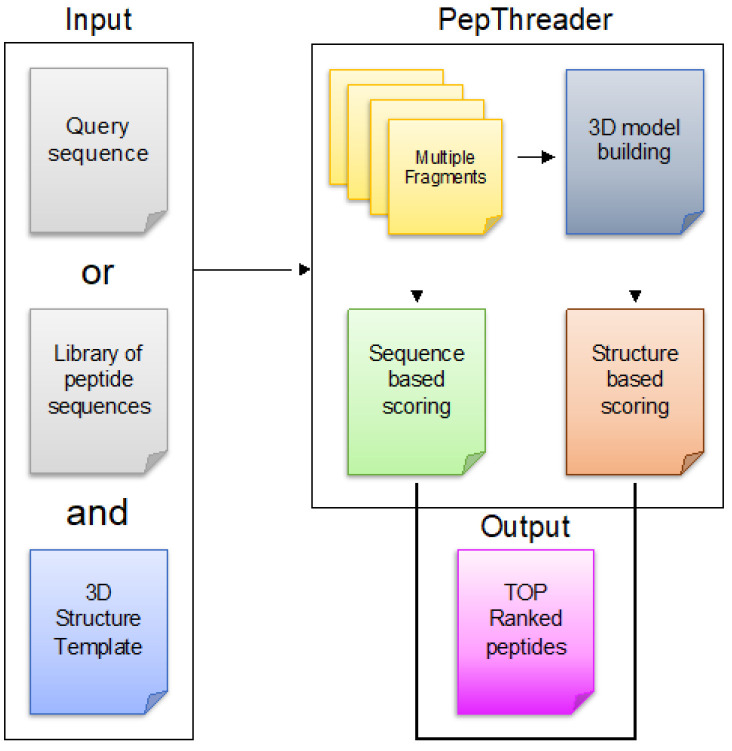
Workflow of the PepThreader algorithm. PepThreader receives as input a protein sequence or a library of peptides in FASTA format and a template protein–peptide complex in PDB format. The sequence is divided into multiple peptides and the latter are ranked according to an alignment matrix score. The best ranking peptides are then modeled on the template protein and 3D structure models are generated for these peptides. A second structure-based ranking is then calculated by energy scoring functions. The output consists of a series of peptides in complex with their receptor, ranked by sequence-based and energy-based scores.

The overall flow chart of PepThreader is described in Figure 1. PepThreader receives as input a library of peptides derived from a protein sequence provided by the user, or a library derived from putative binding peptides selected by the user, and it gives as output a pool of peptides that could possibly bind a target protein, providing also 3D models of the predicted protein–peptide complexes.

The software is entirely written in Python [33] and freely available. It makes use of widespread Python libraries for data analysis, such as Biopython [34] and Pandas [35]. The code can be found at https://github.com/cgasb/PepThreader.git (accessed on 20 January 2022).

### 2.1. Required Inputs

The first release of PepThreader provides at the moment a command-line usage only (in future releases, a graphical interface and a web server are foreseen). As described next, different inputs are required to carry out the analysis: a PDB structure (hereinafter the “receptor”) in complex with a cognate peptide; the sequence of a protein that is known to interact with the receptor, in FASTA format; the number of models (“decoys”) built for each peptide fragment, extracted from the input sequence; if multiprocessing is required, the number of cores used for the analysis; and an output directory where 3D models should be stored. Every fragment of the query sequence will be associated to a specific subdirectory containing the peptide sequence, 3D models, and scoring results.

### 2.2. Multiple Peptides Fragments Generation and Scoring by Alignment Matrices

Once the input files are loaded, the sequence of the protein is divided into multiple fragments (“*k-mers*”), based on the length *k* of the template peptide. The algorithm makes use of a “sliding window” across the input sequence and divides it into multiple fragments. Optionally, fragments of different length *k* can be generated. The obtained peptide *k-mers* are then ranked according to a score that is computed making use of sequence matrices. This step of the algorithm, which is mainly sequence-based (see below), requires low computational costs, thus resulting in an extremely fast process.

At the moment, six different sequence scoring matrices are implemented in PepThreader: BLOSUM62 [36], QU3 [37], their “GALAXY” versions (BLOSUM62-GALAXY and QU3-GALAXY, similarly as described in the GalaxyPepDock algorithm [26]), and “Modified” versions (BLOSUM62-M and QU3-M; see below). This modification allows for the adding of binding site characteristics (derived from the template complex) to the simple alignment matrix, and to obtain a score that does not depend exclusively on sequence similarity. Indeed, a major flaw of sequence scoring matrices is the lack of information regarding the biochemical and biophysical properties of the chemical environment of each amino acid residue in its folded protein chain [36]. Therefore, the original BLOSUM62 and QU3 matrix scores are modified by a weight that multiplies the number of hydrophobic or ionic protein residues contacting the given peptide residue in the template complex structure by the scoring matrix components with scores > 0. In this way, more emphasis is put on the peptide residues contributing to interactions with the receptor, than on other residues during peptide alignment.

This concept can be expressed in mathematical terms as follows:

*Galaxy B*(*j*,*j*′) = {1 + [*N_inter_*(*j*) × *ΘB*(*j*,*j*′)]} × *B*(*j*,*j*′) (1)

where *B*(*j*,*j*′) is the original value of the scoring matrix, *N_inter_* is the number of interacting hydrophobic or ionic protein residues with the peptide residue *j* in the template complex structure, and *Θ* is a modifier that assume a value of 0 if *B*(*j*,*j*′) is ≤0, and 1 if *B*(*j*,*j*′) is >0. Hydrophobic (or ionic) contacts are defined when at least two heavy atoms of protein–peptide residue pairs are within a certain distance threshold (default is 5.0 Å; [26]). In “GALAXY” matrices [26], the modification is applied only on evolutionary conserved residue pairs (*B*(*j*,*j*′) is >0). On the other hand, we also tested the performance of these modified matrices by setting *Θ* always to 1. In this way, we also considered those cases where the two residue pairs are not well conserved during evolution (*B*(*j*,*j*′) ≤ 0). To discriminate these two conditions in PepThreader, they are hereinafter named “GALAXY” and “Modified” matrices, respectively.

As a result, the algorithm ranks all the peptides and returns a list of *k-mers* that could possibly bind the receptor. Optionally (see below), another structure-based ranking of the output peptides can be carried out, using scoring functions derived from the obtained 3D models, with the aim of obtaining a more accurate prediction.

### 2.3. Template Based 3D Modeling of Protein–Peptide Complexes

3D structures of protein–peptide complexes are then modeled making use of the MODELLER [38] package. For every protein–peptide complex, the algorithm generates a number of alternative decoys, each of which is scored according to different scoring functions (described below). In order to accelerate the process, the modeling part can be parallelized in multiple cores whose number is set by the user.

### 2.4. Scoring and Ranking of Protein–Peptide Complexes with 3D Scoring Functions

After the first sequence-based ranking, a protein–peptide complex can be optionally subjected to a reranking based on 3D scoring functions. Only the top-scoring peptides coming from the first ranking (with a threshold score decided by the user) are considered for the second ranking. In this way, the time spent in model generation and assessment is reduced. Indeed, though computationally much more expensive, such structure-based reranking is particularly accurate at discriminating binding from non-binding peptides. The aim of the reranking procedure is therefore to improve the ranking accuracy to spot the true-positive binding peptides. At the moment, three different structure-based scoring functions are implemented in PepThreader: Soap potentials [39]; DOPE score [40]; and Voronoi-derived scores, such as the *voromqa* and *voromqa* interaction score [41]. This class of statistical potentials can be described as particular functions that associate a score (also defined as “statistical potential energy”) to a distance between an atom pair in a molecule. The value of this score is proportional to the frequency of these distances observed in a training set of experimentally determined structures. In this way, the different types of interactions (polar, hydrophobic, and electrostatic) are implicitly computed before measuring the peptide–protein docking score.

### 2.5. Benchmark Dataset

PepThreader has been tested using a dataset of protein–peptide complexes derived from different databases: PixelDB [42], LEAD PEP [43], PEP-PRO [44], PEPTIDB [32], and ACCLUSTER [45]. Each entry of the dataset is composed of pairs of protein–peptide complexes, with a single protein receptor and two different peptides. While the first protein–peptide complex is used as template and is needed to carry out the analysis, the other is used to verify the ability of PepThreader to spot the true-positive peptide among the set of fragments generated from the full-length sequence. In assembling the final dataset, composed of 292 entries (Supplementary File S1), different requirements were taken into consideration: peptides length should be between 4 and 14 residues; sequence identity between peptide pairs should be ≤80% (this filter was applied to remove pairs with highly similar sequences); the ratio of the length of the shortest peptide in the pair over the length of the longer one should be >0.6 (because peptides should have similar length); and the binding site of the receptor should be the same for both peptides. With those filters, most entries were composed by structures derived from MHC complexes, which are the most common protein–peptide complexes stored in the PDB. This observation led us to reduce the number of pairs in the dataset by randomly selecting a maximum number of pairs (in this case five) for every receptor. To highlight a possible correlation between the performance of PepThreader and the physico-chemical properties of the peptides, the values of the hydrophobicity index of each peptide in our dataset were computed and correlated to the rank of each peptide obtained with the SOAP-PEP scoring function. This analysis does not show any type of correlation (Pearson correlation coefficient = −0.07; data not shown).

The accuracy tests were carried out considering the number of times PepThreader could spot the experimentally determined peptide among the 50 top-scoring peptides only with sequence scoring matrices, or also with structure-based scoring functions (top 25).

### 2.6. Comparison of Target and Template Peptide–Receptor Complexes

In order to evaluate the level of structural divergence between target and template peptides bound to the same receptor, we used the root mean square deviation (*RMSD*) metric. When we considered a pair of target and template peptide–receptor structures, we first rigidly superimposed the receptor chains of the two structures. We then computed the *RMSD* between the Cα atoms of the equivalent residues of the peptides (equivalences are attributed according to a sequence-based alignment between the peptides). The *RMSD* value is computed as follows:

(2) $$RMSD=\sqrt{\frac{1}{N}\overset{N}{\underset{i=1}{\sum }}d_{i}{}^{2}}$$

where *N* is the number of equivalent Cα atom pairs and *d_i_* is the distance in space of the *i*—the pair after the superimposition of the receptor chains. The lower the *RMSD* value is, the higher is the level of structural similarity between the conformations of the two peptides when bound to the receptor.

## 3. Results and Discussion

### 3.1. Overview of PepThreader

Given a protein sequence (or library of peptides), PepThreader predicts (“spots”) which fragments are able to bind to a given protein receptor. In order to identify the binding peptide(s), PepThreader integrates two different ranking schemes, a sequence-similarity-based score, and a structure-based forcefield. The first ranking method is used to filter out peptides that, according to their sequences, are unlikely to bind to the protein target. This preliminary step helps to reduce the number of potential binders to be tested, saving the high computational time that would be required to 3D model hundreds of fragments for a protein complex. Optionally, the top-scoring peptide sequences can be then 3D modeled in complex with the protein target and used to compute an interaction score that is derived from a structure-based metric (see the section named “Scoring and Ranking of protein–peptide complexes with 3D scoring functions”). At the end of both schemes, the algorithm returns a ranked list of peptides that could possibly bind the template protein.

### 3.2. Properties of Query Sequences and Target–Template Pairs

**Figure 2:**
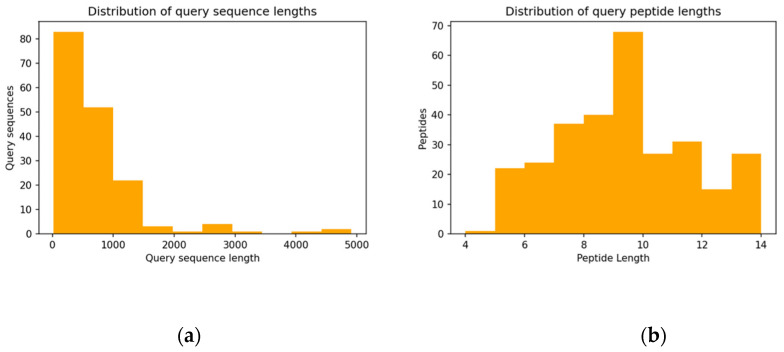
Distribution of the query sequence length and query peptides length: (**a**) *n* = 292, *mean* = 742, *std* = 738; (**b**) *n* = 292, *mean* = 9.0, *std* = 2.3.

The right-skewed distribution of the full-length query sequences shows that most of the queries have a length that is between 100 and 1500 amino acids, with an average length of 742. Only seven sequences are over 2500 amino acids long. Regarding the query peptides, which were selected for having a length between 4 and 14 residues (Figure 2b), a sequence length of nine residues are the most frequently found.

**Figure 3:**
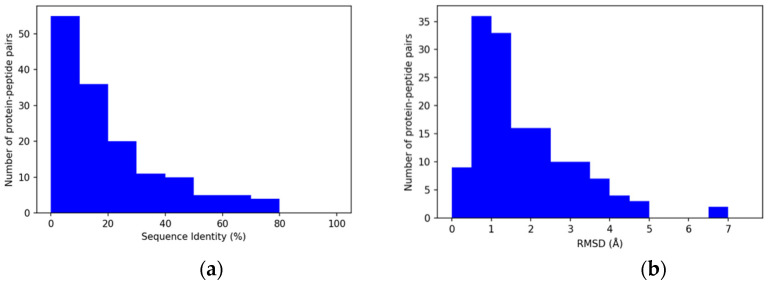
Distribution of sequence identity and *RMSD* in complex pairs: (**a**) Sequence identity in protein–peptide pairs. As shown in the histogram, the identity goes from 0.0% to a maximum value of 77.8% (*n* = 146, *mean* = 18.2%, *std* = 19.6%); (**b**) distribution of the Cα *RMSD* between the peptides in the protein–peptide complex pairs, expressed in Angstroms (Å). Values are between 0.4 Å and 6.8 Å (*n* = 146, *mean* = 1.8 Å, *std* = 1.3 Å).

The target–template complexes were analyzed by comparing the template and the query peptides for their sequence identity and structural similarity. The two classes of peptides are in most cases quite different and their sequence identity is usually between 0% and 30% (Figure 3a). Highly similar peptide sequences would be easily top-ranked only according to sequence similarity-based scores. Therefore, peptides with a sequence identity higher than 80% were excluded from the dataset to avoid an overestimation of the PepThreader performance.

The distribution of the Cα RMSDs (Figure 3b), computed between the target and template peptides once superposed on the binding pocket of their receptor, showed values with a mean of 1.82 Å, and a maximum value of 6.79 Å. This distribution is consistent with the idea that different peptides are bound to the receptor protein with a similar binding mode (e.g., occupying the same binding pocket and having the same N- to C-terminal orientation along the pocket).

### 3.3. Sequence Similarity-Based Score

Regarding the sequence similarity-based score, we relied on different evolutionary matrices (Table 1) for benchmarking purposes. In this phase, an output of 50 peptides was chosen as it seemed an appropriate compromise between the number of peptides to be evaluated in the next structure-based phase, and the computational time required to 3D model them. Matrices without any specific modification did not generally perform well on our benchmark dataset. For example, the unmodified QU3 and BLOSUM62 matrices spotted the true binder peptides in the top 50 predicted peptide sequences with an accuracy of 66% and 63%, respectively. On the other hand, the “GALAXY”-modified versions of the BLOSUM62 and QU3 matrices slightly increased the accuracy of the predictions. The modified BLOSUM62 matrix (“BLOSUM62-M”; see “Methodology” section) resulted in being the more efficient alignment matrix in recognizing the experimental binding peptide as the best ranking (top one in the 24% of the total cases). This percentage reasonably increases when the output is not limited to the top-ranking peptide, but to a list of best ranking ones. For example, the accuracy increases from 24% to 49.0%, 62.0%, 69.9%, and to 71.6%, when considering as output 10, 25, 50, and 75 peptides, respectively. According to these results, we decided to choose the BLOSUM62-M as the default for sequence scoring purposes in PepThreader.

The relationship between the length of the query sequence and the performance of the BLOSUM62-M is reported in Table 2. This analysis showed that the algorithm performs worst in query sequences with a length between 500 and 1000 residues, while, surprisingly, it seems to perform better on those sequences that have an extremely long length (2000–5000). This result was somewhat unexpected, as the number of fragments is lower in the case of shorter sequences. Likely, the nature of the peptide sequence and the possible presence of consensus sites positively influence the results. Furthermore, as expected, an improvement of the performance is observed with an increase of sequence identity (Table 3).

### 3.4. Structure-Based Score

The top 25 peptide sequences, identified after the initial sequence similarity-based scoring scheme, were then used to generate 3D models that were subsequently ranked based on their interaction energy computed with four different scoring functions.

To compute only rank improvements over the first sequence-based scoring scheme, the total number of protein–peptide complexes was reduced by removing from the analysis the experimental peptides already ranked among the top 25 by BLOSUM62. As reported in Table 4**,** among the tested scoring functions, SOAP-PPI showed the best ability to recognize the true binders (top 10; 55.9%) on the entire benchmark dataset. This value further increased when considering the top 25 peptides (80.0%). SOAP-PPI was also the scoring function with the highest percentage of rank improvements (53.5%) and the only one to have a positive average rank change. It is important to mention that those changes are greatly conditioned by the gap between the rankings of the two scoring schemes, e.g., a significant negative result can highly influence the average value. Indeed, if considering only the positive ranking improvements, the use of scoring functions increased on average the rank value of the experimental binding peptides of 12 positions for the SOAP-PEP scoring function, and 15 positions for the voromqa scoring function. Therefore, the mean positive improvement in ranking obtained with structure-based scoring functions justifies their use in refining the predictions of PepThreader.

To get additional insights into the second structure-based scoring scheme of PepThreader, the performance of all the scoring functions was compared to the *RMSD* of each target–template complex. To this purpose, the dataset was divided into three different classes, according to the *RMSD* value between the pairs of protein–peptide complexes: “Low” (L; *RMSD* from 0.0 Å to 1.5 Å); “Mid” (M; *RMSD* from 1.5 Å to 3.0 Å); and “High” (H; *RMSD* above 3.0 Å). Low values in *RMSD* between templates can be partially related to the sequence identity percentage of the peptides, which is 0.32, 0.23, and 0.12 for “Low”, “Mid”, and “High” *RMSD* values, respectively.

Since the structure-based score depends on the modeled peptide conformation, a high performance for the algorithm in top-ranking queries with low *RMSD* values is expected. The change in the performance when the structure-based score is computed, rather than the sequence-based only, showed, for all scoring functions, a performance increment when queries with high *RMSD* values were analyzed (Table 5). Therefore, the usage of a structure-based score can be of utmost importance in the identification of the true binders, even when the analyzed peptides have a different binding mode with respect to the one used as a template. It is likely that the low amelioration of the efficiency when analyzing queries with low *RMSD* values underlies the ability of the algorithm in identifying the true binders already in the sequence-based ranking, due a correlation between high sequence identity and low *RMSD* values.

### 3.5. An Application of PepThreader: The ESCRT-I Interacting Peptides

**Figure 4:**
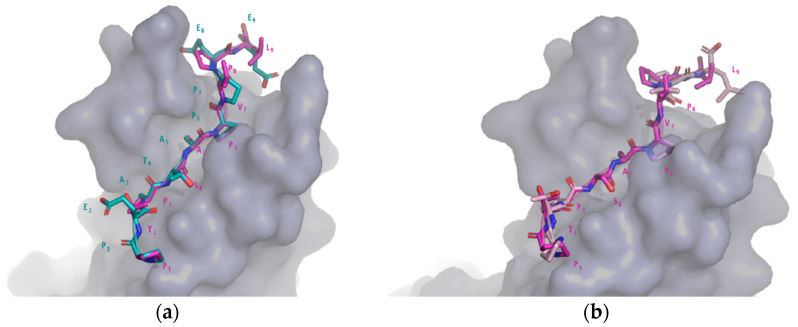
Results of PepThreader usage for spotting a TSG101 peptide binder. (**a**) 3D model of the complex between the top-ranked peptide of HRS and TSG101 (peptide “PTPSAPVPL” highlighted in magenta), superimposed to the 3D structure used as a template (PDB code: 3OBX, peptide “PEATAPPEE” highlighted in light blue); (**b**) the same 3D model of (**a**) superimposed to the corresponding crystal structure (PDB code: 3OBQ).

As a practical application of the use of PepThreader, in this section we illustrate its ability to spot a HRS (a mammalian master molecule in vesicular transport and protein sorting)-derived peptide that binds the TSG101 subunit of ESCRT (Human Endosomal Sorting Complex Required for Transport), and for which the tridimensional structure of the complex was solved (PDB: 3OBQ; [46]; Figure 4a). This interaction is a promising potential therapeutic target for protein–peptide interactions inhibitors, since ESCRT is also required for HIV-1 budding [47]. The crystal structure of TSG101 in complex with a HIV-1 peptide “PEATAPPEE” was used as template (PDB: 3OBX; [46]).

The query HRS protein sequence (UniProt code: O14964; sequence length: 777 residues), which contains the peptide of interest (“PTPSAPVPL”), was fragmented into 776 short nonapeptides. According to the sequence-based score of PepThreader (BLOSUM62-M), the true binder was initially ranked in position 20. Continuing the analysis with the reranking, thus applying the structure-based score (SOAP-PEP), the true binder was the top ranked (position 1). Therefore, PepThreader was able to spot the correct peptide and rank it first out of 776 potential hits. The final *RMSD* between the obtained 3D model and the corresponding crystal structure was 0.89 Å (Figure 4b).

### 3.6. An Application of PepThreader: Identifying the Critical Site of Bora to Bind and Activate Aurora-A

**Figure 5:**
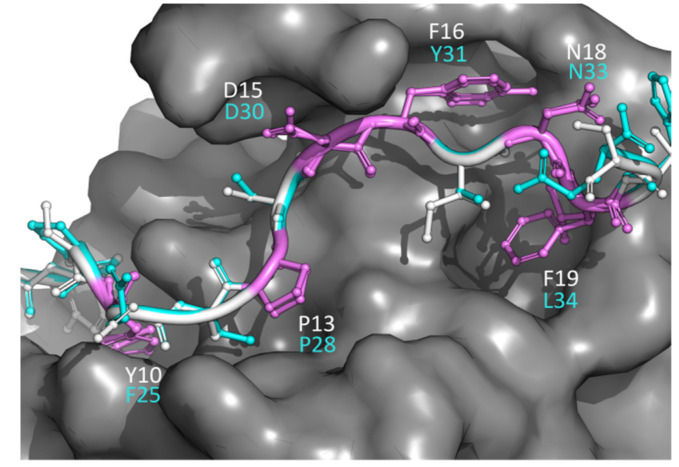
Results of PepThreader usage for spotting the M1 peptide binder of Bora to Aurora-A. The structural superposition of Bora (cyan) motif M1 with TPX2 motif (white) responsible for binding to Aurora-A (gray surface) is shown. The essential residues in TPX2 responsible for binding Aurora-A and the corresponding residue numbers in Bora are highlighted.

As a second application of the use of PepThreader, in this section we illustrate its ability to identify and model the key determinants of human Bora (Protein Aurora Borealis) required for the activation of the kinase Aurora-A at the onset of mitosis, as recently described by reference [48]. In the latter, it has been demonstrated that a minimal fragment of Bora, encompassing residues 18–120, supports the Aurora-dependent phosphorylation of Plk1 in vitro. Within this region, using mutagenesis on Bora^18–120^, a motif described as “M1” (residues 25–34 in Bora) has been pinpointed as a key determinant of Aurora-A binding and activation [48]. Therefore, we relied on the crystal structure of Aurora-A in complex with the first 43 residues of TPX2 (a well-characterized allosteric activator of Aurora-A; PDB: 1OL5 [49]) to assess the ability of PepThreader to spot the M1 motif within the Bora^18–120^ region given as input. According to both scores of PepThreader, i.e., sequence-based BLOSUM62-M and structure-based SOAP-PEP, the M1 motif was first ranked out of 92 generated peptide fragments. The obtained 3D model highlighted key “hot-spots” of binding between Aurora-A and Bora that resemble the same interactions observed for the Aurora-A/TPX2 complex (Figure 5). In particular, the hydrophobic (Tyr10, Phe16, and Phe19) and polar (Asp15, Asn18) interactions of TPX2 with Aurora-A are conserved in Bora (Phe25, Phe31, Leu34, Asp30, and Asn33).

## 4. Conclusions

PepThreader is a novel algorithm that has the potential to “spot” peptides binding to a given target from a full-length sequence or a library of peptides given as input. At the state of the art, only few software can do this kind of analysis, often requiring long times and high computational power. Therefore, developing a tool that can recognize binding peptides, and also generate a 3D structure model of the interaction with the target protein, can give researchers great help on studying PPIs and PpIs.

The algorithm has an ambitious goal, because discriminating the true binding peptide(s) over a library of hundreds of potential binders on average can be challenging. In the current release, PepThreader is not always capable of reaching this goal, but it can predict within a high probability that the true binding peptide(s) could be within a limited subset. On the other hand, PepThreader comes also with the ability to model the 3D structure of the protein–peptide complex, and this feature can be used to overcome the simple sequence similarity criterion and predict whether a single point mutation is sufficient to prevent the binding of a peptide (e.g., a bulky sidechain replacing a small one would result in steric clashes that hamper the binding).

In any case, the future development prospects of PepThreader foresee several strategies to improve its performance. For example, regarding the sequence-based ranking, the alignment matrices could be modified to take into account the secondary structures of both template and query peptides. Indeed, if the template and the target peptides have a similar secondary structure, the alignment score could be rewarded, leading to a higher rank for these peptides. Moreover, it is reasonable to consider that recent machine learning approaches for protein structure prediction (e.g., AlphaFold2 [50]) will be tailored for modeling protein–peptide interactions. Though MODELLER, the current modeling engine implemented into PepThreader, is sufficiently accurate to this purpose, using advanced statistical modeling approaches could produce even more accurate predictions.

In conclusion, PepThreader is a unique tool that is able at the same time to spot a binding peptide within a full-length sequence involved in PPI and model its structure within the receptor. Currently, there are no other tools able to perform these kinds of predictions with such a simple approach and short times of analysis.

## Figures and Tables

**Table 1:** The table shows the performance of several alignment matrices in PepThreader in terms of percentages of true binders identified in several ranking ranges.

Matrix	Top 1	Top 10	Top 25	Top 50	Top 75
BLOSUM62	57/292 (19.5%)	143/292 (49.0%)	165/292 (56.5%)	194/292 (66.4%)	209/292 (71.6%)
BLOSUM62-M	70/292 (24.0%)	143/292 (49.0%)	181/292 (62.0%)	204/292 (69.9%)	209/292 (71.6%)
BLOSUM62 GALAXY	69/292 (23.6%)	143/292 (49.0%)	185/292 (63.3%)	200/292 (69.8%)	217/292 (74.3%)
QU3	48/292 (16.4%)	117/292 (40.0%)	154/292 (52.7%)	183/292 (62.7%)	211/292 (72.2%)
QU3-M	65/292 (22.2%)	138/292 (47.3%)	177/292 (60.6%)	200/292 (68.4%)	214/292 (73.3%)
QU3 GALAXY	61/292 (20.9%)	139/292 (47.6%)	178/292 (61.0%)	201/292 (68.8%)	214/292 (73.3%)

**Table 2:** Relationship between the query sequence length and the efficiency of BLOSUM62-M. The table reports the algorithm efficiency in terms of percentage of true binders identified.

Matrix	Top 10	Top 25	Top 50	Top 75
Length 0–500	69/138 (50.0%)	92/138 (66.6%)	101/138 (73.2%)	106/138 (76.8%)
Length 500–1000	30/79 (38.0%)	38/79 (48.1%)	45/79 (57.0%)	49/79 (62.0%)
Length 1000–2000	32/58 (55.2%)	39/58 (67.2%)	42/58 (72.4%)	44/58 (75.9%)
Length 2000–5000	12/17 (70.6%)	12/17 (70.6%)	16/17 (94.1%)	16/17 (94.1%)

**Table 3:** Relationship between sequence identity of template–query peptides and efficiency of modified BLOSUM62. The table reports the algorithm efficiency in terms of percentage of true binders identified.

Matrix	Top 1	Top 10	Top 25	Top 50
SeqId 0–25	37/162 (22.8%)	56/162 (34.6%)	75/162 (46.3%)	86/162 (53.1%)
SeqId 25–50	79/102 (77.4%)	98/102 (96.1%)	101/102 (99.0%)	101/102 (99.0%)
SeqId 50–75	18/19 (94.7%)	18/19 (94.7%)	19/19 (100%)	19/19 (100%)
SeqId 75–100	9/9 (100%)	9/9 (100%)	9/9 (100%)	9/9 (100%)

**Table 4:** Ranking improvements and efficiency of the scoring functions. In brackets are shown the percentages of the ratios and the average rank change calculated on positive results.

Scoring Function	Number of Improved	Average Rank Change	Top 10 after Reranking	Top 25 after Reranking
DOPE	42/127 (33.1%)	−5.9 (13)	42/127 (33.1%)	87/127 (68.5%)
SOAP-PPI	68/127 (53.5%)	0.5 (13)	71/127 (55.9%)	102/127 (80.0%)
SOAP-PEP	62/127 (48.8%)	−0.1 (12)	69/127 (54.3%)	102/127 (80.0%)
voromqa	51/127 (40.2%)	−1.6 (15)	57/127 (44.8%)	96/127 (75.6%)
voromqa-int	50/127 (39.4%)	−2.2 (13)	56/127 (44.1%)	97/127 (76.4%)

**Table 5:** Ranking improvements and performance of the scoring functions. In brackets are shown the percentages of the ratios and the average rank change calculated in positive results. The results for each scoring function are divided in groups according to *RMSD* values (L; *RMSD* from 0.0 Å to 1.5 Å); “Mid” (M; *RMSD* from 1.5 Å to 3.0 Å); and “High” (H; *RMSD* above 3.0 Å).

Scoring Function	N. Improved	Average SeqId	Average Rank Change	Top 10 after Reranking	Top 25 after Reranking
DOPE (L)	23/77 (29.9%)	0.23	−6.5 (13.4)	42/127 (33.1%)	87/127 (68.5%)
DOPE (M)	11/36 (30.6%)	0.16	−5.9 (16.6)	71/127 (55.9%)	102/127 (80.0%)
DOPE (H)	8/14 (57.1%)	0.13	−1.6 (8.2)	69/127 (54.3%)	102/127 (80.0%)
SOAP-PPI (L)	43/77 (55.8%)	0.27	0.13 (9.9)	57/127 (44.8%)	96/127 (75.6%)
SOAP-PPI (M)	17/36 (47.2%)	0.22	1.1 (15.5)	56/127 (44.1%)	97/127 (76.4%)
SOAP-PPI (H)	8/14 (57.1%)	0.09	1.1 (9.0)	7/14 (50.0%)	11/14 (78.6%)
SOAP-PEP (L)	36/77 (46.8%)	0.27	−0.8 (10.5)	44/77 (57.1%)	62/77 (80.5%)
SOAP-PEP (M)	18/36 (50.0%)	0.20	1.0 (15.2)	16/36 (44.4%)	29/36 (80.6%)
SOAP-PEP (H)	8/14 (57.1%)	0.10	1.0 (8.4)	9/14 (64.3%)	11/14 (78.6%)
voromqa (L)	31/77 (40.3%)	0.22	−1.7 (12.6)	39/77 (50.6%)	61/77 (79.2%)
voromqa (M)	13/36 (36.1%)	0.19	−2.3 (18.2)	13/36 (36.1%)	24/36 (66.7%)
voromqa (H)	7/14 (50.0%)	0.11	0.71 (13.6)	5/14 (35.7%)	11/14 (78.6%)
voromqa-int (L)	28/77 (36.4%)	0.22	−2.2 (13.6)	37/77 (48.1%)	60/77 (77.9%)
voromqa-int (M)	15/36 (41.6%)	0.19	−3.2 (13.8)	12/36 (33.3%)	25/36 (69.4%)
voromqa-int (H)	7/14 (50.0%)	0.11	2.92 (16.1)	7/14 (50.0%)	12/14 (85.7%)

## Data Availability

The open-source code of PepThreader, together with installation instructions can be found at https://github.com/cgasb/PepThreader.git (accessed on 20 January 2022).

## Supplementary Materials

The following are available online at https://www.mdpi.com/article/10.3390/biom12020201/s1, File S1: Dataset of 292 entries used to benchmark PepThreader.
